# Supplementary material for: Determinants of shingles vaccine acceptance in the United Kingdom
Source: PLoS One. 2019 Aug 1;14(8):e0220230. doi: 10.1371/journal.pone.0220230 (PMC6675065; doi:10.1371/journal.pone.0220230)
Supplement: S1 Table — (DOCX) [file pone.0220230.s001.docx]

**S1 Table. Determinants of Shingles Vaccination**

|  | **Bivariate Analysis**  **(N=501)** | | | | | | **Multivariable Model**  **(N=348)** | |
| --- | --- | --- | --- | --- | --- | --- | --- | --- |
|  | **Vaccinated (N=344)** | | | **Unvaccinated (N=147)** | | **p-value** | **OR** | **95% CI** |
|  | **n (%)** | **Mean (SD)** | | **n (%)** | **Mean (SD)** |  |  |  |
| **SOCIO-DEMOGRAPHICS** | | | | | | | | |
| Sex | 344 | |  | 147 |  |  |  |  |
| Male | 143 (41.6) | | - | 58 (39.5) | - | 0.663 | 1.0 |  |
| Female | 201 (58.4) | | - | 89 (60.5) | - |  | 1.4 | 0.8-2.6 |
| GP’s geographical location | 306 | |  | 126 |  |  |  |  |
| Urban | 191 (62.4) | | - | 69 (54.8) | - | 0.140 | NA | NA |
| Rural | 115 (37.6) | | - | 57 (45.2) | - |  |  |  |
| Ethnic group | 331 | |  | 143 |  |  |  |  |
| White | 326 (98.5) | | - | 143 (100.0) | - | 0.140 | NA | NA |
| Non-white | 5 (1.5) | | - | 0 (0.0) | - |  |  |  |
| Living situation | 331 | |  | 143 |  |  |  |  |
| Living with family or friends | 17 (5.1) | | - | 8 (5.6) | - | 0.252 | NA | NA |
| Living with wife, husband, or partner | 113 (34.1) | | - | 51 (35.7) | - |  |  |  |
| Living in a care home or assisted accommodation | 191 (57.7) | | - | 75 (52.4) | - |  |  |  |
| Living alone | 2 (0.6) | | - | 3 (2.1) | - |  |  |  |
| Living alone with the help of a carer | 2 (0.6) | | - | 4 (2.8) | - |  |  |  |
| Prefer not to say | 6 (1.8) | | - | 2 (1.4) | - |  |  |  |
| Level of education | 331 | |  | 140 |  |  |  |  |
| University + College | 112 (33.8) | | - | 43 (30.7) | - | 0.619 | 1.0 |  |
| Primary school | 21 (6.3) | | - | 10 (7.1) | - |  | 0.7 | 0.2-3.2 |
| High school | 187 (56.5) | | - | 79 (56.4) | - |  | 1.1 | 0.5-2.2 |
| Prefer not to say | 11 (3.3) | | - | 8 (5.7) | - |  | 0.3 | 0.1-1.5 |
| Professional/caring activity | 316 | |  | 132 |  |  |  |  |
| Still working (full time or part time) | 9 (2.8) | | - | 0 (0.0) | - | 0.072 | 1.0 |  |
| Retired but active (volunteer work, caring for family) | 70 (22.2) | | - | 24 (18.2) | - |  |  |  |
| None | 216 (68.4) | | - | 103 (78.0) | - |  | 0.6 | 0.3-1.2 |
| Prefer not to say | 21 (6.6) | | - | 5 (3.8) | - |  | 3.1 | 0.6-14.9 |
| Household income | 326 | |  | 135 |  |  |  |  |
| High (£36,400 – £51,999 - £52,000 or over) | 17 (5.2) | | - | 2 (1.5) | - | 0.160 | 1.0 |  |
| Low (up to £5,199 to £15,600 – £20,799) | 109 (33.4) | | - | 42 (31.1) | - |  | 0.4 | 0.1-2.1 |
| Medium (£20,800 – £25,999 - £31,200 – £36,399) | 44 (13.5) | | - | 15 (11.1) | - |  | 0.5 | 0.1-2.9 |
| Prefer not to say | 156 (47.9) | | - | 76 (56.3) | - |  | 0.3 | 0.1-2.0 |
| **HEALTH** | | | | | | | | |
| Assessment of own health as “good” | 320 | |  | 136 |  |  |  |  |
| No^1^ | 66 (20.6) | | - | 38 (27.9) | - |  | 1.0^1^ |  |
| Yes | 254 (79.4) | | - | 98 (72.1) | - | 0.088 | 1.0 | 0.5-1.9 |
| Self-reported current conditions | 344 | |  | 147 |  |  |  |  |
| No condition | 119 (34.6) | | - | 38 (25.9) | - | 0.057 | 1.0 |  |
| Any conditions | 225 (65.4) | | - | 109 (74.1) | - |  | 0.7 | 0.4-1.4 |
| History of shingles | 344 | |  | 145 |  |  |  |  |
| No^1^ | 239 (69.5) | | - | 81 (55.9) | - | <0.001 | 1.0^1^ |  |
| I don't know/remember^1^ | 17 (4.9) | | - | 3 (2.1) | - |  |  |  |
| Yes | 88 (25.6) | | - | 61 (42.1) | - |  | 0.4**γ | 0.2-0.7 |
| Extent of participation in health decisions | 332 | |  | 140 |  |  |  |  |
| My GP always makes decisions for me | 46 (13.9) | | - | 14 (10.0) | - | 0.494 | 1.0 |  |
| Like to know options but GP decides | 74 (22.3) | | - | 36 (25.7) | - |  | 0.5 | 0.2-1.5 |
| GP and I make decisions together | 138 (41.6) | | - | 59 (42.1) | - |  | 0.5 | 0.2-1.4 |
| I make decisions after GP advice | 70 (21.1) | | - | 27 (19.3) | - |  | 0.5 | 0.1-1.6 |
| I always make own decisions | 4 (1.2) | | - | 4 (2.9) | - |  | 0.4 | 0.0-5.0 |
| **KNOWLEDGE** | | | | | | | | |
| Knowledge about shingles | 336 | | 3.4 (1.74) | 143 | 3.4 (1.98) | 0.878 |  |  |
| Limited knowledge (1 to 4) | 256 (76.2) | | - | 102 (71.3) | -- | 0.262 | 1.0 |  |
| Good knowledge (5 to 7) | 80 (23.8) | | - | 41 (28.7) | - |  | 1.1 | 0.5-2.6 |
| Individual true knowledge of shingles (8a-8d)^2^ | 338 | | 2.6 (1.10) | 144 | 2.4 (1.24) | 0.270 | 0.8 | 0.6-1.1 |
| Knowledge about shingles vaccine | 339 | | 2.7 (1.63) | 144 | 2.5 (1.82) | 0.103 |  |  |
| Limited knowledge (1 to 4) | 296 (87.3) | | - | 122 (84.7) | - | 0.445 | 1.0 |  |
| Good knowledge (5 to 7) | 43 (12.7) | | - | 22 (15.3) | - |  | 0.5 | 0.2-1.5 |
| **CONSTRUCTS** | | | | | | | | |
| Perceived susceptibility | 332 | | 3.6 (1.27) | 142 | 3.8 (1.24) | 0.036 | 1.0 | 0.8-1.3 |
| I consider myself to be at risk of developing shingles | 330 | | 3.5 (1.51) | 142 | 3.5 (1.52) | 0.781 |  |  |
| If I had shingles, I would feel more ill than other people my age with shingles would feel | 327 | | 3.4 (1.76) | 139 | 3.9 (1.65) | 0.005 |  |  |
| If I had shingles, I would feel vulnerable to other illnesses or complications | 325 | | 3.8 (1.69) | 136 | 4.0 (1.66) | 0.205 |  |  |
| If I have had chickenpox, I need the shingles vaccine^3^ | 317 | | 4.6 (1.67) | 137 | 4.4 (1.46) | 0.134 |  |  |
| Perceived severity | 337 | | 4.9 (1.26) | 143 | 5.0 (1.19) | 0.775 | 1.2 | 0.9-1.6 |
| If I developed shingles, it could be very painful | 333 | | 5.9 (1.63) | 143 | 5.8 (1.74) | 0.802 |  |  |
| If I developed shingles, I could become seriously ill | 331 | | 4.4 (1.60) | 142 | 4.4 (1.46) | 0.825 |  |  |
| Shingles could prevent me from carrying out simple daily activities | 332 | | 4.7 (1.77) | 143 | 4.8 (1.71) | 0.582 |  |  |
| If I developed shingles, I could suffer long-term complications (e.g. pain, eye problems, etc.) | 330 | | 4.8 (1.70) | 141 | 4.9 (1.68) | 0.521 |  |  |
| Perceived benefits | 327 | | 5.6 (1.11) | 136 | 5.3 (1.03) | 0.002 | 1.0 | 0.8-1.4 |
| The shingles vaccine can protect me from getting shingles | 323 | | 5.6 (1.62) | 135 | 5.4 (1.45) | 0.236 |  |  |
| The shingles vaccine can reduce the severity of the symptoms if I develop shingles | 322 | | 5.8 (1.38) | 135 | 5.5 (1.40) | 0.024 |  |  |
| The shingles vaccine is effective | 320 | | 5.5 (1.35) | 134 | 4.9 (1.28) | <0.001 |  |  |
| Perceived barriers | 332 | | 2.9 (1.08) | 138 | 3.6 (0.99) | <0.001 | 0.7 | 0.5-1.0 |
| The shingles vaccine could make me ill | 324 | | 3.1 (1.57) | 134 | 3.7 (1.40) | <0.001 |  |  |
| The shingles vaccination is painful | 327 | | 2.1 (1.65) | 135 | 3.0 (1.62) | <0.001 |  |  |
| The shingles vaccine could give me shingles | 318 | | 2.6 (1.57) | 136 | 3.3 (1.58) | <0.001 |  |  |
| The shingles vaccine is a new vaccine that has not yet been tested enough | 322 | | 3.3 (1.48) | 136 | 3.7 (1.44) | 0.003 |  |  |
| I do not need the shingles vaccination if I have had shingles in the past | 322 | | 3.3 (1.68) | 132 | 3.9 (1.69) | <0.001 |  |  |
| I am worried about having too many vaccines | 324 | | 3.1 (1.95) | 134 | 3.6 (1.98) | 0.008 |  |  |
| Practical barriers and Facilitators | 329 | | 6.2 (1.40) | 129 | 5.8 (1.62) | 0.008 | 1.0 | 0.8-1.3 |
| It was easy for me to get to a place (e.g. clinic, doctor's surgery, etc.) where I could have the shingles vaccination | 328 | | 6.2 (1.53) | 128 | 5.8 (1.75) | 0.013 |  |  |
| I had enough free time to get the shingles vaccination | 324 | | 6.3 (1.43) | 128 | 5.9 (1.64) | 0.006 |  |  |
| The fact that the shingles vaccination requires only a single visit to the doctor's surgery is important to me^3^ | 326 | | 5.8 (1.69) | 135 | 5.4 (1.70) | 0.008 |  |  |
| Self-efficacy | 316 | | 5.9 (1.80) | 127 | 5.2 (2.16) | <0.001 | 1.2*γ | 1.0-1.4 |
| There was no reason I could not get the shingles vaccination if I had wanted to | 316 | | 5.9 (1.80) | 127 | 5.2 (2.16) | <0.001 |  |  |
| Perceived control of disease | 323 | | 2.7 (1.56) | 130 | 3.5 (1.54) | <0.001 | 0.7*γ | 0.6-0.9 |
| I can avoid developing shingles even without the shingles vaccine | 323 | | 2.7 (1.56) | 130 | 3.5 (1.54) | <0.001 |  |  |
| Trust | 329 | | 6.2 (1.39) | 136 | 5.9 (1.37) | 0.064 | 1.2 | 0.9-1.5 |
| I trust my GP’s advice about the shingles vaccine | 328 | | 6.3 (1.45) | 136 | 6.0 (1.42) | 0.107 |  |  |
| I trust the National Health Service’s (NHS) recommendations on the shingles vaccine | 326 | | 6.1 (1.47) | 135 | 5.8 (1.50) | 0.059 |  |  |
| **OTHER SOCIO-PSYCHOLOGICAL FACTORS** | | | | | | | | |
| Did your GP or nurse offer you the shingles vaccination (through a letter, phone call, text message or during a visit)? | 321 | |  | 139 |  |  |  |  |
| I don't know/remember^1^ | 35 (10.9%) | | - | 16 (11.5%) | - | <0.001 | 1.0^1^ |  |
| No^1^ | 66 (20.6%) | | - | 56 (40.3%) | - |  |  |  |
| Yes | 220 (68.5%) | | - | 67 (48.2%) | - |  | 2.3*γ | 1.1-4.7 |
| Did your GP or nurse tell you about shingles? | 315 | |  | 131 |  |  |  |  |
| I don't know/remember^1^ | 47 (14.9%) | | - | 13 (9.9%) | - | <0.001 | 1.0^1^ |  |
| No^1^ | 126 (40.0%) | | - | 79 (60.3%) | - |  |  |  |
| Yes | 142 (45.1%) | | - | 39 (29.8%) | - |  | 0.7 | 0.3-1.4 |
| Do you know anyone who has had shingles? | 324 | |  | 141 |  |  |  |  |
| I don't know/remember^1^ | 6 (1.9%) | | - | 4 (2.8%) | - | 0.740 | 1.0^1^ |  |
| No^1^ | 51 (15.7%) | | - | 20 (14.2%) | - |  |  |  |
| Yes | 267 (82.4%) | | - | 117 (83.0%) | - |  | 0.8 | 0.4-1.6 |
| Do you know anyone who has had the shingles vaccination? | 325 | |  | 137 |  |  |  |  |
| I don't know/remember^1^ | 19 (5.8%) | | - | 5 (3.6%) | - | <0.001 | 1.0^1^ |  |
| No^1^ | 151 (46.5%) | | - | 92 (67.2%) | - |  |  |  |
| Yes | 155 (47.7%) | | - | 40 (29.2%) | - |  | 1.6 | 0.8-3.2 |
| Did anyone, among your vaccinated relatives or friends, advise you to have the shingles vaccination? | 325 | |  | 135 |  |  |  |  |
| I don't know/remember^1^ | 29 (8.9%) | | - | 6 (4.4%) | - | 0.016 | 1.0^1^ |  |
| No^1^ | 242 (74.5%) | | - | 117 (86.7%) | - |  |  |  |
| Yes | 54 (16.6%) | | - | 12 (8.9%) | - |  | 1.6 | 0.6-4.4 |
| *Max-rescaled R-Square (pseudo-R^2^)* |  | |  |  |  |  | 0.3220 | |

CI = Confidence Interval; OR = Odds ratio; *p ≤ 0.05; γ direction and significance of effect corroborated in sensitivity analysis.

^1^ Multivariable model reference category is “Other than yes”. It includes “No”, “I don’t know/remember” and missing.

^2^ A correct response for Q8a is False, Q8b-Q8d is True. Each of the correct response represents a score of 1.

^3^ To improve Cronbach’s alpha, (internal consistency), Q11a and Q11d were removed from Perceived susceptibility and Practical barriers and Facilitators’ constructs, respectively. The dependent variables were pre-selected items based on correlation coefficient (Pearson coefficient≤│0.80│ for quantitative variables* and Cohen Kappa ≤0.75 for categorical variables^#^. Variance inflation factor and conditional index were determined to assess collinearity and robustness of model (conditional index >30 and variance inflation factor <10).

^[[1]](#footnote-1)^

1. * Mukaka MM. A guide to appropriate use of Correlation coefficient in medical research. Malawi Med J. 2012;24(3):69-71.

   ^#^ Mandrekar JN. Measures of interrater agreement. J J Thorac Oncol. 2011;6(1):6-7. [↑](#footnote-ref-1)
