# Supplementary material for: Determinants of shingles vaccine acceptance in the United Kingdom
Source: PLoS One. 2019 Aug 1;14(8):e0220230. doi: 10.1371/journal.pone.0220230 (PMC6675065; doi:10.1371/journal.pone.0220230)
Supplement: S1 Appendix — (DOCX) [file pone.0220230.s002.docx]

**S1 Appendix. Individual questionnaire**

| Item | Response categories |
| --- | --- |
| 1) Today’s date: | DD MM YYYY |
| 2) Are you filling in the questionnaire yourself? | Yes / no |
| 3) Do you consider yourself to be in good health? | Yes / no |
| 4) Do you currently have one or more of the following conditions? (Multiple select) | - Heart or vascular disease - Lung disease - Diabetes - Cancer - Arthritis or mobility problems - Stress, anxiety or depression - Other conditions – please specify: |
| 5) For each of the following questions, please tick (✓) your answer:   1. Have you ever had shingles? 2. Do you regularly have a flu vaccination? 3. Have you ever had the shingles vaccination? | Yes / no / I don’t know/remember |
| 6) In general, to what extent do you participate with your GP in making decisions about your health? (Single select) | - My GP always makes decisions for me. - I like to know the options available, but I still let my GP make decisions for me. - My GP and I make decisions together. - I make decisions for myself after considering the advice of my GP. - I always make my own decisions, independently of the advice of my GP. |
| 7) Please circle the number that best represents how much you know about shingles | Scale 1-7: very little / a lot |
| 8) For each of the following statements, please tick (✓) the answer that best represents your opinion:   1. Shingles can be caught from someone else who has shingles. 2. Shingles can lead to long-lasting, severe pain. 3. The chance of developing shingles increases with age. 4. Shingles is caused by the same virus that causes chickenpox. | Yes / no / I don’t know/remember |
| 9) For each of the following statements about shingles, please circle the number that best represents your opinion (where 1 is ‘I strongly disagree’ and 7 is ‘I strongly agree’):   1. I consider myself to be at risk of developing shingles. 2. If I had shingles, I would feel more ill than other people my age with shingles would feel. 3. If I had shingles, I would feel vulnerable to other illnesses or complications. 4. If I developed shingles, it could be very painful. 5. If I developed shingles, I could become seriously ill. 6. Shingles could prevent me from carrying out simple daily activities. 7. g) If I developed shingles, I could suffer long-term complications (e.g. pain, eye problems, etc.). | Scale 1-7: I strongly disagree / I strongly agree |
| 10) Please circle the number that best represents how much you know about the shingles vaccine (where 1 is ‘Very little’ and 7 is ‘A lot’): | Scale 1-7: very little / a lot |
| 11) For each of the following statements about the shingles vaccine, please circle the number that best represents your opinion (where 1 is ‘I strongly disagree’ and 7 is ‘I strongly agree’):   1. If I have had chickenpox, I need the shingles vaccine. 2. The shingles vaccine can protect me from getting shingles. 3. The shingles vaccine can reduce the severity of the symptoms if I develop shingles. 4. The fact that the shingles vaccination requires only a single visit to the doctor's surgery is important to me. 5. The shingles vaccine is effective. 6. The shingles vaccine could make me ill. 7. The shingles vaccination is painful. 8. The shingles vaccine could give me shingles. 9. The shingles vaccine is a new vaccine that has not yet been tested enough. | Scale 1-7: I strongly disagree / I strongly agree |
| 12) For each of the following questions, please tick (✓) your answer:   1. Did your GP or nurse offer you the shingles vaccination (through a letter, phone call, text message or during a visit)? 2. If you received a letter about the shingles vaccination from your GP, was it in a language that you understand? 3. Did your GP or nurse tell you about shingles? 4. Do you know anyone who has had shingles? 5. Do you know anyone who has had the shingles vaccination? 6. Did anyone, among your vaccinated relatives or friends, advise you to have the shingles vaccination? 7. Where did you learn about the shingles vaccine? (Multiple Select) | For questions **a) - f)** : Yes / no / I don’t know/remember  For question **g**) :   - NHS website or printed NHS materials - Doctor’s surgery - TV/Radio - Social media (Facebook, Twitter, etc.) - Newspapers/Magazines - Non-NHS websites (e.g. alternative medical websites) - Manufacturer’s website/leaflet - Other – please specify: - None of the above |
| 13) Which of the following best describes your ethnic group? (Single select) | - White/British/Irish/Other White - Black/African/Caribbean/Black British - Mixed/Multiple Ethnic Groups (e.g. White & Black, White & Asian, etc.) - Asian/Asian British (e.g. Indian, Pakistani, Bangladeshi, Chinese, etc.) - Other Ethnic Group (e.g. Arab) - Prefer not to say |
| 14) Which of the following best describes your current living situation? (Single select) | - Living with family or friends - Living alone - Living with your wife, husband or partner - Living alone with the help of a carer - Living in a care home or assisted accommodation - Prefer not to say |
| 15) What is the highest level of education that you have completed? (Single select) | - Primary school/Junior school - High school/Secondary school - College - University - Prefer not to say |
| 16) Are you taking part in any of the following? (Multiple select) | - Full-time paid employment - Part-time paid employment - Voluntary work - Caring for family members or friends   (IF for longer than 1 hour per day)   - None of the options - Prefer not to say |
| 17) What is your annual household income (or your own annual income, if you live by yourself)? (Single select) | - Up to £5,199 - £5,200 – £10,399 - £10,400 – £15,599 - £15,600 – £20,799 - £20,800 – £25,999 - £26,000 – to £31,199 - £31,200 – £36,399 - £36,400 – £51,999 - £52,000 or over - Prefer not to say |

**Table.** Physician questionnaire

| Item | Response categories |
| --- | --- |
| Demographics and Clinical experience | |
| 1 a) Age (in years): | <30 / 30-39 / 40-49 / 50-59 / 60-69 / ≥ 70 |
| 1 b) Gender: | Male / female |
| 1 c) Location: | Urban (> 10,000 residents) / Rural (≤ 10,000 residents) |
| 1 d) Year of medical qualification: | Year |
| 1 e) Number of physicians in your practice: | Free number entry field |
| 1 f) Number of nurses in your practice: | Free number entry field |
| 1 g) Number of other personals in your practice: | Free number entry field |
| Vaccine recommendations and activities at your practice | |
| 2) For each of the following statements regarding vaccine recommendations for your elderly patients (65 years or older), please circle the option that best represents your opinion:   1. Seasonal Influenza vaccine 2. Pneumococcal vaccine 3. Zoster vaccine 4. Diphtheria, Tetanus, Polio (+/- Pertussis) vaccine if history of vaccination is unclear | Scale 1-7: strongly against / strongly recommend |
| 3) Do you have internal procedures/guidelines for vaccination (e.g. checklist, vaccination procedure, etc.) at your practice for your elderly patients (65 years or older)? | Yes / no / prefer not so say |
| 4) Does your day-to-day workload allow you enough time to provide vaccinations information or recommendations to your elderly patients (65 years or older)? | Yes / no / prefer not to say |
| 5) Do you consider you have enough internal staff (nurses, residents, other colleagues, etc.) at your practice to provide vaccinations information or recommendations to your elderly patients (65 years or older)? | Yes / no / prefer not to say |
| Zoster Vaccine | |
| 6) Have there been communication campaigns regarding Zoster vaccination (e.g. local radio/TV spot, local newspapers advertisement, etc.) in your area? | Yes / no / I don’t know/remember |
| 7) Do you have informational materials at your practice about Zoster vaccination for your patients (e.g. leaflets, posters, etc.)? | Yes / no / I don’t know/remember |
| 8) Please circle the number which best represents how much you are familiar with the Zoster vaccination campaign and related informational materials: | Scale 1-7: not familiar at all / very familiar |
| 9) For each of the following statements about Zoster and the Zoster vaccine, please circle the option that best represents your opinion:   1. Zoster is a serious disease that requires preventive actions for elderly. 2. Zoster represents a significant economic burden for elderly. 3. There is enough information about the duration of protection of the Zoster vaccine. 4. The Zoster vaccine is safe. 5. The Zoster vaccine is effective. 6. I think that my patients do not need the Zoster vaccine because Zoster is rare. 7. I think that my patients should not get the Zoster vaccine because they have received too many vaccines. 8. My patients think they do not need the Zoster vaccine. 9. My patients are worried about getting the Zoster vaccine. 10. The fact that the Zoster vaccine only requires a single injection is an advantage. 11. The fact that the Zoster vaccine has an established real life experience from various countries is an advantage. | Scale 1-7: I strongly disagree / I strongly agree |
