# Supplementary material for: Determinants of shingles vaccine acceptance in the United Kingdom
Source: PLoS One. 2019 Aug 1;14(8):e0220230. doi: 10.1371/journal.pone.0220230 (PMC6675065; doi:10.1371/journal.pone.0220230)
Supplement: S2 Appendix — (DOCX) [file pone.0220230.s003.docx]

**S2 Appendix. Physician questionnaire**

| Item | Response categories |
| --- | --- |
| Demographics and Clinical experience | |
| 1 a) Age (in years): | <30 / 30-39 / 40-49 / 50-59 / 60-69 / ≥ 70 |
| 1 b) Gender: | Male / female |
| 1 c) Location: | Urban (> 10,000 residents) / Rural (≤ 10,000 residents) |
| 1 d) Year of medical qualification: | Year |
| 1 e) Number of physicians in your practice: | Free number entry field |
| 1 f) Number of nurses in your practice: | Free number entry field |
| 1 g) Number of other personals in your practice: | Free number entry field |
| Vaccine recommendations and activities at your practice | |
| 2) For each of the following statements regarding vaccine recommendations for your elderly patients (65 years or older), please circle the option that best represents your opinion:   1. Seasonal Influenza vaccine 2. Pneumococcal vaccine 3. Zoster vaccine 4. Diphtheria, Tetanus, Polio (+/- Pertussis) vaccine if history of vaccination is unclear | Scale 1-7: strongly against / strongly recommend |
| 3) Do you have internal procedures/guidelines for vaccination (e.g. checklist, vaccination procedure, etc.) at your practice for your elderly patients (65 years or older)? | Yes / no / prefer not to say |
| 4) Does your day-to-day workload allow you enough time to provide vaccinations information or recommendations to your elderly patients (65 years or older)? | Yes / no / prefer not to say |
| 5) Do you consider you have enough internal staff (nurses, residents, other colleagues, etc.) at your practice to provide vaccinations information or recommendations to your elderly patients (65 years or older)? | Yes / no / prefer not to say |
| Zoster Vaccine | |
| 6) Have there been communication campaigns regarding Zoster vaccination (e.g. local radio/TV spot, local newspapers advertisement, etc.) in your area? | Yes / no / I don’t know/remember |
| 7) Do you have informational materials at your practice about Zoster vaccination for your patients (e.g. leaflets, posters, etc.)? | Yes / no / I don’t know/remember |
| 8) Please circle the number which best represents how much you are familiar with the Zoster vaccination campaign and related informational materials: | Scale 1-7: not familiar at all / very familiar |
| 9) For each of the following statements about Zoster and the Zoster vaccine, please circle the option that best represents your opinion:   1. Zoster is a serious disease that requires preventive actions for elderly. 2. Zoster represents a significant economic burden for elderly. 3. There is enough information about the duration of protection of the Zoster vaccine. 4. The Zoster vaccine is safe. 5. The Zoster vaccine is effective. 6. I think that my patients do not need the Zoster vaccine because Zoster is rare. 7. I think that my patients should not get the Zoster vaccine because they have received too many vaccines. 8. My patients think they do not need the Zoster vaccine. 9. My patients are worried about getting the Zoster vaccine. 10. The fact that the Zoster vaccine only requires a single injection is an advantage. 11. The fact that the Zoster vaccine has an established real-life experience from various countries is an advantage. | Scale 1-7: I strongly disagree / I strongly agree |
