# Supplementary material for: Determinants of shingles vaccine acceptance in the United Kingdom
Source: PLoS One. 2019 Aug 1;14(8):e0220230. doi: 10.1371/journal.pone.0220230 (PMC6675065; doi:10.1371/journal.pone.0220230)
Supplement: Attachment — Submitted filename: R2R.docx [file pone.0220230.s004.docx]

**Reviewer #1**: The study is original, relevant and well written.

Table 2 is too long and could be changed. I suggest that author may choose most important comparisons to present in this table. Example: perceived susceptibility / severity / benefits may be enough. I think all sub-items are not necessary and make the table complex and boring. Authors can choose most important findings to keep in a shorter table 2.

**Authors’ response**: Thank you for your suggestion. We have incorporated those changes in the manuscript.

**Reviewer #2**: The paper addresses a relevant and interesting topic such as the evaluation of determinants of shingles vaccine acceptance in the UK. This survey is interesting being UK the first European country to introduce a national immunization program for shingles. For this reason, this experience could be relevant for other countries that now are starting or implementing this vaccination.

The manuscript is well written, very clear and complete.

I would suggest to describe and comment on the pilot test (please see page 4 lines 86-87)

Figure 2. The grey scale is not very clear (at least on my pc)

**Authors’ response:** Thank you for your suggestions. We have incorporated those changes in the manuscript and changed the color shade of Figure 2.

I look forward to your final decision and I am available should you need further assistance.

Yours sincerely,

Ivy Xiaoyan LU

Associate Director, Vaccines-CORE

MSD Vaccins 162, avenue Jean Jaurès 69007 LYON | France

[ivy.xiaoyanlu@gmail.com](mailto:ivy.xiaoyanlu@gmail.com)

[xiaoyan.lu@msd.com](mailto:xiaoyan.lu@msd.com)

T: +33 (0)7 89558468/M: +33 (0)4 37284
